# What Models and Tools can Contribute to a Better Understanding of Brain Activity?

**DOI:** 10.3389/fnetp.2022.907995

**Published:** 2022-07-18

**Authors:** Marc Goodfellow, Ralph G. Andrzejak, Cristina Masoller, Klaus Lehnertz

**Affiliations:** ^1^ Living Systems Institute, University of Exeter, Exeter, United Kingdom; ^2^ Department of Information and Communication Technologies, University Pompeu Fabra, Barcelona, Spain; ^3^ Department of Physics, Universitat Politecnica de Catalunya, Barcelona, Spain; ^4^ Department of Epileptology, University of Bonn Medical Centre, Bonn, Germany; ^5^ Helmholtz Institute for Radiation and Nuclear Physics, University of Bonn, Bonn, Germany; ^6^ Interdisciplinary Center for Complex Systems, University of Bonn, Bonn, Germany

**Keywords:** neuroscience, network, dynamical systems, data analysis, brain

## Abstract

Despite impressive scientific advances in understanding the structure and function of the human brain, big challenges remain. A deep understanding of healthy and aberrant brain activity at a wide range of temporal and spatial scales is needed. Here we discuss, from an interdisciplinary network perspective, the advancements in physical and mathematical modeling as well as in data analysis techniques that, in our opinion, have potential to further advance our understanding of brain structure and function.

## 1 Introduction

Over the last two decades, investigations of brain network structure and function—w.r.t. both physiologic and pathophysiologic conditions—has gained strong impetus from the success made in the quantitative analysis of complex networks (Bullmore and Sporns, 2009; Bullmore and Sporns, 2012; Stam, 2014; Bassett and Sporns, 2017; Lynn and Bassett, 2019). Accompanied by an ever increasing technology that allows access to brain structure and function at various spatial and temporal scales, neuroscientific research (both basic science and clinically-oriented research) has demonstrated a remarkable success in improving our knowledge of brain network structure and function. Nevertheless, despite worldwide effort, huge gaps remain in our understanding of how networks at various scales give rise to emergent dynamics, i.e., brain function and dysfunction.

In this Perspective article, we argue that further progress on these problems would benefit from effort invested in truly cross-disciplinary research (Wickson et al., 2006; Woolf, 2008). That is, research in one discipline that not only utilises results coming from the other but also works back to understand and leverage the reciprocal contribution. As well as uncovering potentially novel, fruitful approaches, this might serve as a means to bring different communities closer together. Our aim here is to highlight some approaches from our own research experience for which this reciprocal bridging could be advanced.

We first concentrate on the sub-disciplines Data Analysis and (mathematical) Modeling that both are often be assumed to be mature. Although it was repeatedly shown that advancements in one sub-discipline can help to balance disadvantages in the other, both these sub-disciplines face serious limitations when it comes to the brain’s structure-function relationship. We argue that progress here could benefit from a greater emphasis on experiments to validate models and enhanced model calibration. Closer cross-disciplinary links in the cycle of model refinement and checking would facilitate progress here.

We then offer two other concrete examples of neuroscience-inspired research that could make a reciprocal contribution back to neuroscience. These are from the (seemingly unrelated) sub-disciplines—research into excitable optical systems (Photonic Neurons) as well as into the co-existence of synchronization and desynchronization (Chimera States). We suggest that these largely experimental physics, and applied mathematics based research directions have a lot to offer back to the investigation of the function of the brain, and that progress could be made in the short to medium term.

## 2 Data Analysis

Data-driven approaches continue to contribute to improve our understanding of function and structure of the complex system that is the brain. Current approaches are typically based on different concepts from mathematics, physics, or computer science and provide various indices that aim at characterizing different (linear or nonlinear) properties of some dynamics as wells as of properties of interactions (strength, direction, functional form) between two or more (sub-)systems (Lehnertz et al., 2014; Lehnertz et al., 2017 and references therein). Approaches that characterize properties of interactions form the basis (so called “functional” and “effective” connectivity) of a data-driven construction of functional brain networks (Eguıluz et al., 2005; Bullmore and Sporns, 2009; Bullmore and Sporns, 2012; Stam, 2014; Bassett and Sporns, 2017; Lynn and Bassett, 2019). These approaches are backed up with a variety of imaging techniques (Sporns, 2011; Fornito et al., 2015; Rockland, 2015; Fornito et al., 2019; Sotiropoulos and Zalesky, 2019; Sarwar et al., 2021; Yeh et al., 2021; Gosak et al., 2022) that aim at characterizing the so called “structural” connectivity, which is often considered ground truth and underlying constraint on “functional”/“effective” connectivity. Structural information is mostly derived from magnetic-imaging-based techniques such as diffusion tensor imaging (DTI). Although widely employed, DTI has severe limitations as it does not allow to recognize crossing fibers and it fails to identify/visualize fibers along and within cortical surface (Mori and Zhang, 2006). By now, there are no commonly accepted means to validate DTI findings.

Time series analysis approaches to characterize the strength of (pairwise) interactions are often assumed to be mature as they allow one to characterize interactions between sequences of amplitudes, phases, frequencies (or mixtures of the latter), interactions between representations of the dynamics in state space, between information flows, and even interactions between stochastic dynamics. Time series analysis approaches to characterize the direction of interactions, however, need further development. Findings that can be achieved with many of the currently available techniques require careful interpretation (ideally with the help of appropriate surrogate techniques yet to be developed) as they touch upon the notoriously difficult issue of identifying causal relationships (Mayr, 1961; Laland et al., 2011). Time series analysis approaches to characterize the functional form of an interaction have been developed only recently, and are mostly restricted to phase-based interactions (Stankovski et al., 2017).

With many of the aforementioned techniques to derive characteristics of pairwise interactions from observations, interactions are assumed to be constant (at least during the investigated time interval). This assumption might not be fully justified for the inherently nonstationary system brain, notwithstanding the wide range of endogenous and exogenous biological rhythms impacting differently on its structure and function (Lehnertz et al., 2021). Another and long-standing issue (e.g., Zentgraf, 1975) centers around identifying and characterizing higher-order interactions, i.e., interactions that cannot be reduced to pairwise interactions. Although this issue recently has received increasing attention (e.g., Schneidman et al. (2006); Benson et al. (2016); Lambiotte et al. (2019); Battiston et al. (2020); Skardal and Arenas (2020); Battiston et al. (2021); Lacasa et al. (2021); Zhang et al. (2021); Majhi et al. (2022)), there are by now no time series analysis techniques suited to sufficiently characterize higher-order interaction from observations of brain dynamics.

Developments along the aforementioned lines need to be accompanied by necessary advancements in network theory, ranging from an improved characterization of weighted and directed networks to the characterization of hypergraphs and simplicial complexes. With an eye on applications, there is a strong need for concepts and indices that allow one to reliably compare and quantify differences in brain networks inferred from data (Mheich et al., 2020). Eventually, future developments need to respect the multi-scale character of the brain’s structure and functions, ranging from single cells to larger brain regions, from localized oscillations to scale-free dynamics, and with time scales from a few minutes to several days and weeks and beyond (cf. Gosak et al. (2018)).

## 3 Models

Many mathematical models have been developed and studied at different spatial scales in the brain, i.e., from ion channels, synapses and neuronal membranes through to the electrical activity of the whole brain, as recorded by EEG. Modelling at the single neuron scale, or smaller, could be considered to be the most tractable, since relevant closed systems (e.g., synapses) can be experimentally isolated, and parameters of the system (e.g., time scales) can be measured directly (e.g., Lee et al., 2015). At the other end of the spatial scale, which we refer to as mesoscopic, or macroscopic, we aim to model and understand the dynamics of regions of brain tissue that contain many thousands of neurons and other cells (Deco et al., 2008; Breakspear, 2017). This scale of measurement is important since it is the level at which human brain dynamics, and whole brain dynamics consisting of integrated brain systems, can most often be recorded (e.g., using fMRI, EEG and MEG in humans). Given the complexity of interacting processes that give rise to this kind of data, mathematical models are crucial for understanding the brain at this scale (Breakspear, 2017).

Macroscopic brain models can be roughly grouped according to the different kinds of assumption they make in their derivation (Deco et al., 2008; Breakspear, 2017). Despite their differences, most make use of building blocks that represent, in their simplest forms, interactions between excitatory and inhibitory neuronal populations. The parameter values that govern macroscopic dynamics in these models are often based upon findings at the microscopic level (Rall, 1967; Lopes da Silva et al., 1974; Freeman, 1975). Of course simplifications have to be made, and assumptions can be made explicit. However, macroscopic brain dynamics emerge from underlying complex systems in tissues that comprise varied, heterogeneous cells and molecules. This means that macroscopic model parameters cannot be constrained solely by knowledge at the neuronal level. We should therefore study large ranges of parameters in our models in order to fully explore which model settings can plausibly recreate features of the data (Ferrat et al., 2018), and we should expect there to be multiple parameter regions that are plausible (Hartoyo et al., 2019). This could mean different inferences from the same data and model, depending upon which region of parameter space is studied. In addition, further research is required to better understand the link between microscopic mechanisms and parameters of macroscopic brain models. This can be done by comparing the dynamics of models at different scales (Wendling et al., 2012) and by using perturbations in experimental systems to test the validity of macroscopic model assumptions and predictions. Examples of this kind of research are surprisingly lacking (Freeman, 1975; Moran et al., 2011), particularly studies making use of current methodologies like optogenetics (Bernal-Casas et al., 2017). All of these endeavours will require developments in how we explore the parameters of (often high-dimensional) macroscopic models, and how we compare model output to data. Standard frameworks that exist, such as dynamic causal modelling (Friston et al., 2019), are not designed to fully explore large, minimally constrained parameter spaces. They often rely on linearisation of models (West et al., 2021) and linear data features such as the power spectrum.

Macroscopic models could also help us understand the longer time scales of brain dynamics such as those governing the recurrence of seizures in epilepsy (Suffczynski et al., 2004; Lytton, 2008; Goodfellow et al., 2011; Maturana et al., 2020) or fluctuations in the alpha rhythm (Freyer et al., 2011). From the dynamical systems perspective, mechanisms exist that can give rise to a repertoire of long term, fluctuating behaviour such as different types of intermittency (Pomeau and Manneville, 1980; Platt et al., 1993; Velazquez et al., 1999; Goodfellow et al., 2011), heteroclinic switching (Rabinovich et al., 2014) and multistability (Golos et al., 2016). Determining if these mechanisms are responsible for a given recording of long term brain dynamics will be challenging, since few such recordings exist (Weisdorf et al., 2019; Duun-Henriksen et al., 2020; Maturana et al., 2020; Zaer et al., 2021) and none are yet publically available. Further, we do not yet know how to rule out alternative mechanisms that could equally well account for observations like critical slowing down (Milanowski and Suffczynski, 2016; Wilkat et al., 2019; Maturana et al., 2020; Hagemann et al., 2021). Other models—including physiological models—that have some of the alternative dynamic mechanisms named above, can presumably yield a repertoire of long term dynamics, depending upon their parameterisation (Goodfellow et al., 2012 unpublished).

## 4 Photonic Neurons

Photonic neurons are optical systems with neuron-like output signals (e.g., Coomans et al., 2011; Nahmias et al., 2013; Dolcemascolo et al., 2018; Tiana-Alsina et al., 2019; Robertson et al., 2021). A lot of research is nowadays focused on building photonic neurons able to accurately mimic the way neurons process information, and to demonstrate that such photonic neurons can perform information processing tasks such as sensing, classification, or logic operations using efficient, noise-robust neural coding mechanisms. Interest is driven by the fact that photonics neurons are not only energy-efficient, but also, ultra-fast, and have potential to process information orders of magnitude faster than biological neurons, or current silicon technology (e.g., Shastri et al., 2021 and references therein).

A key ingredient of neuronal dynamics is excitability, and for implementing photonics neurons, it is crucial to identify suitable excitable optical systems. The dynamics of an excitable system has the following characteristics: 1) An input below a threshold results in a small amplitude response; 2) an input above the threshold elicits a spike; 3) a stronger input does not change the shape of spike; 4) two well separated super-threshold inputs elicit two spikes; 5) if the two inputs are too close in time (the interval between them being smaller than a refractory period) the second input does not elicit a spike. These characteristics have been observed in inexpensive, energy-efficient semiconductor (diode) lasers, under optical perturbations (e.g., Giudici et al., 1997; Wieczorek et al., 2002; Garbin et al., 2017; Robertson et al., 2020). However, when comparing the (experimentally recorded) laser response to weak periodic inputs, with a neural response (simulated with a simple neuron model), some significant statistical differences have been uncovered (Tiana-Alsina et al., 2019). It is still not clear which types of neurons the different excitable laser systems may represent, and more research is needed for performing an in-depth comparison of the statistical properties of spike sequences (optical and neural) emitted under different types of external inputs. For example, it was recently shown that neuronal ensembles could encode weak inputs using symbolic spike patterns (Masoliver and Masoller, 2020), and arrays of excitable lasers may be used for testing this possibility.

Photonic neuromorphic computing is the research field that aims at using photonic neurons and neuronal mechanisms of information coding and processing for implementing ultra-fast photonic artificial neural networks (ANNs). In recent years, impressive advances have been made in improving the performance of photonic ANNs by developing efficient training methods, expanding the number of nodes and integrating them into silicon chips (e.g., Antonik et al., 2020; Lugnan et al., 2020). However, important challenges remain: how to identify conditions in which the spikes emitted by photonic neurons genuinely represent neuronal spikes, and how to implement in photonic neurons the efficient mechanisms by which neurons encode, transmit and process weak external inputs in noisy environments, exploiting nonlinear phenomena such as excitability, bistability and stochastic resonance (Barbay et al., 2000; Marino et al., 2002).

Single neurons and neuronal populations use different coding mechanisms depending on the type of input, and neuronal responses at different timescales may encode different features of an input (e.g., Quiroga and Panzeri, 2013). An important open challenge is to understand how these coding mechanisms can be implemented in photonic neurons, where various noise sources (optical, electrical, thermal, and mechanic) can play a role similar to neural noise in biological neurons.

Advances in this field can be expected to also have an impact in neuroscience, since photonic neurons that emit optical spikes that are very similar (and perhaps not even distinguishable from) neural spikes will allow to perform controlled experiments, to advance the understanding of the generation and propagation of spiking activity in neural networks.

Research on nonlinear photonic systems may also allow to advance other problems that are relevant to neuroscience. An example is how to infer reliable indicators of an approaching transition to a different, and potentially dangerous, dynamical regime (e.g., an epileptic seizure). In terms of dynamical systems, such transitions may result from a time-varying parameter that crosses a bifurcation point. In this context, lasers have been used as “toy models” because they allow to perform controlled experiments, to test the capability of new time series analysis tools for providing reliable indicators alerting of incoming bifurcations (e.g., Masoller et al., 2015; Marconi et al., 2020, where the abrupt switching of the polarization of the emitted light and the crossing of the laser threshold were used to analyze the suitability of a well-known indicator—critical slowing down—to provide reliable warning of the dynamical transition).

## 5 Chimera States

The term chimera states refers to the co-existence of synchronization and desynchronization in networks of coupled dynamics. These peculiar states were first described in ring networks of non-locally coupled phase oscillators (Kuramoto and Battogtokh, 2002; Abrams and Strogatz, 2004). In this classical model, all oscillators are identical and the coupling is the same for all oscillators. Accordingly, when one moves along the extension of the ring network, the structure remains the same everywhere. The network has translational symmetry. Intuition might therefore suggest that the dynamics should be qualitatively the same for all oscillators. Either they should all synchronize, or they should all evolve mutually incoherently. However, for some ranges of the network parameters, this expectation is wrong, and the symmetry of the network structure is broken by the oscillators’ temporal evolution. After being started with random initial conditions, the oscillators spontaneously segregate into two complementary groups. While one group of oscillators rotates in coherence, the remaining ones perform an erratic motion. This counterintuitive coexistence of synchronization and desynchronization in all-identical elements defines chimera states.

Chimera state networks are an example for a mathematical model system that has a very simple formulation but still generates highly complex dynamics. The study of such simple models can help to understand complex real-world dynamics avoiding the need to resort to overly complicated mechanisms. In 2012, and in the years to follow, it was shown that chimeras can be implemented in experimental setups (e.g., Hagerstrom et al., 2012; Tinsley et al., 2012; Totz et al., 2018; Ebrahimzadeh et al., 2020; Gambuzza et al., 2020), providing first evidence that chimera states do not only exist in mathematical models but can also play a role in real-world dynamics. In parallel, various conceptual links were established between chimeras and a variety of natural and man-made networks outside of experimental labs (e.g., Sakaguchi, 2006; Abrams et al., 2008; Ramlow et al., 2019; Calim et al., 2020; Gerster et al., 2020; Rontogiannis and Provata, 2021). Furthermore, approaches to control chimeras were developed (e.g., Sieber et al., 2014; Bick and Martens, 2015; Omelchenko et al., 2018; Ruzzene et al., 2019; Ruzzene et al., 2020; Vadivasova et al., 2020; Zhang and Dai, 2022).

From early on, there was also a bidirectional transfer of knowledge between research on chimera states and the neurosciences. On the one hand, neuroscience inspired many refinements and generalizations of the mathematical models. Initially, chimera states were mostly studied in isolated single-layer networks. Networks of neurons, however, are typically not isolated but interact with other networks. Therefore, recent work dealt with interactions of chimera states across coupled layers in multilayer networks (e.g., Majhi et al., 2016; Maksimenko et al., 2016; Andrzejak et al., 2017; Andrzejak et al., 2018; Sawicki et al., 2019; Ruzzene et al., 2020; Vadivasova et al., 2020; Rontogiannis and Provata, 2021; Chen et al., 2022). Moreover, various neuron models were used instead of the simple phase oscillators as network nodes (e.g., Sakaguchi, 2006; Hizanidis et al., 2014; Hizanidis et al., 2016; Santos et al., 2017; Chouzouris et al., 2018; Calim et al., 2020; Gerster et al., 2020; Provata and Venetis, 2020; Glaze and Bahar, 2021; Rontogiannis and Provata, 2021; Simo et al., 2021; Zhang and Dai, 2022). In addition, either experimentally obtained real brain connectivity data (e.g., Hizanidis et al., 2016; Santos et al., 2017; Chouzouris et al., 2018; Bansal et al., 2019; Ramlow et al., 2019; Gerster et al., 2020) or basic neuronal connectivity principles (e.g., Rontogiannis and Provata, 2021; Zhang and Dai, 2022) were used to design mathematical model networks capable to show chimera states. Finally, there is growing evidence that results from chimera state networks can contribute to the understanding of fundamental aspects in the balance between synchronization and desynchronization in real neuronal dynamics (e.g., Sakaguchi, 2006; Abrams et al., 2008; Rothkegel and Lehnertz, 2014; Andrzejak et al., 2016; Hizanidis et al., 2016; Chouzouris et al., 2018; Bansal et al., 2019; Gerster et al., 2020; Glaze and Bahar, 2021; Rontogiannis and Provata, 2021).

It is important, however, to keep in mind that even if neuron models and real brain connectivity are used, chimera state network models remain far too simple to be a realistic model of the brain. As a consequence, conceptual links, such as the most prominent one to unihemispheric sleep found in dolphins, birds and other species (Abrams et al., 2008 and references therein), should only be understood as analogies. Neither does a sleeping hemisphere show full synchronization, nor does an awake hemisphere show full desynchronization. Chimera states may nonetheless be helpful for our understanding of this asymmetric behavior of the two brain hemispheres in unihemispheric sleep. In fact, it is perhaps the simplicity of the chimera networks which make them powerful. They allow us to discover basic mechanisms in the interplay of synchronization and desynchronization under well-controlled conditions. In a subsequent step, similar mechanisms can be searched for in neuronal dynamics. In the opposite direction, one can aim to reconstruct peculiarities of neuronal synchronization in a simplified way in chimera networks. On the other hand, advancing the study of other types of synchronization phenomena, such as cluster synchronization, may also contribute to shedding light on brain activity.

## 6 Conclusion

We have outlined challenges in physical and mathematical modeling, and data analysis that should be addressed to tackle deficits in our understanding of the brain as a complex, networked dynamical system. Moreover, we proposed that cross-fertilization of these disciplines, alongside neuroscience, could facilitate significant advances in our knowledge of the brain in health and disease. This could provide exciting opportunities to further advance our understanding of brain functions, brain machine interfaces or neurostimulation. Furthermore, it could be key for the development of effective and personalized treatments for brain disorders which significantly affect the quality of life of the aging world population.

## Data Availability

The original contributions presented in the study are included in the article/Supplementary Material, further inquiries can be directed to the corresponding author.
